# Behind the Illusion: Unmasking the Coercion in Pornography Production

**DOI:** 10.1177/10778012251319300

**Published:** 2025-02-21

**Authors:** Meghan Donevan, Carl Göran Svedin, Inga Dennhag, Linda S. Jonsson

**Affiliations:** 1 Department of Clinical Sciences, Child and Adolescent Psychiatry, 59588 Umeå University, Umeå, Sweden; 2 Department of Social Sciences, 7643 Marie Cederschiöld University, Stockholm, Sweden; 3 Talita, Stockholm, Sweden

**Keywords:** pornography production, sexual exploitation, sexual violence‌, coercion, reflexive thematic analysis

## Abstract

Despite extensive research and public discourse on the effects of pornography on consumers, little attention has been given to the experiences of those filmed for its production. Drawing upon in-depth interviews with 28 individuals filmed for pornography, we explored what pornography represents for them and its implications for their lives. Through reflexive thematic analysis, we identified three main themes: *Coercion behind the illusion of desire*, *A public record*, and *Enduring impacts*. Our findings challenge the notion that pornography predominantly features sexual acts devoid of coercion. We conclude by discussing the legal and practical implications of our findings.

## Introduction

Despite a growing body of research and public discussion focusing on the consequences of pornography for consumers, children, and society at large, almost no attention has been given to its impact on those filmed for its production. Meanwhile, the digital revolution has transformed pornography, reshaping both its production and distribution. Today, virtually anyone can upload, sell, and distribute pornographic content online. Researchers have also noted the digitalization of the sex industry (Cunningham et al., 2018; Jonsson et al., 2015) and the increasingly blurred lines between social media and pornography, where pornography is now exchanged and sold through platforms like Snapchat (Martellozzo & Bradbury, 2021).

Recent investigative journalism efforts and global advocacy campaigns have placed a spotlight on the realities within the pornography industry. For example, a series of articles by Nicholas Kristof in 2020 highlighted the exploitation of children on large pornography platforms, prompting the Canadian parliament to appoint an ethics committee to investigate Pornhub and its parent entity, then Mindgeek and now Aylo (House of Commons Standing Committee on Access to Information, Privacy and Ethics, 2021). In France, 17 men are currently on trial over allegations of rape, procurement, and human trafficking for sexual exploitation in the production of online pornography, with at least 40 victims (Chrisafis, 2023). Such developments emphasize the need to move beyond the exclusive focus on pornography consumption and account for the experiences of those “on the other side of the screen.”

Research incorporating the voices and experiences of individuals filmed for pornography production is limited. Evidence highlights significant risks and adverse health outcomes among this group (e.g., Averdijk et al., 2020; Bishop-Royse et al., 2019; Donevan, 2021; Goldstein et al., 2011; Grudzen et al., 2009, 2011; Javanbakht et al., 2017; King & Evans, 2020), yet little is known about the deeper meanings and implications of their experiences. In other words, what *is* pornography from the perspective of those who appear in its content? What does it represent for them, and what are its broader effects on their lives? This study aims to explore the nature of pornography and its impact as revealed by those with lived experience of being filmed for its production.

### Study Context

This study was conducted in Sweden, a country with relatively liberal social-sexual norms (Person et al., 2016). It was the second country in the world to legalize pornography in 1971. Although Sweden officially prohibits pornography depicting “sexual violence or coercion,” the legislation has, for all intents and purposes, not been enforced (Swedish Ministry of Employment, SOU 2023a).

Sweden strongly prioritizes gender equality and was the first country to adopt the “Nordic model,” otherwise known as the “Equality model” on prostitution in 1999, which penalizes the buyer while decriminalizing and extending support and protection to sellers in prostitution (Månsson, 2017). Seen as a grave form of men's violence against women, prostitution is explicitly included in the government's gender equality goals and prioritized in the fiscal budget. The feminist and women's shelter movements in Sweden have long highlighted the link between prostitution, pornography, and sexual violence. Taking these concerns seriously and considering global revelations about abuse and exploitation in the pornography industry, the Swedish government commissioned an inquiry into the *Protection, Support, and Care of Individuals Subjected to Abuse in the Production or Distribution of Pornography* (Swedish Ministry of Employment, 2023a).

### Theoretical Starting Point and Aim

We employed a broad definition of pornography to capture the wide spectrum of contexts in which pornography is produced, seeking to explore shared meanings and implications across its various forms. Defined as “images, videos, and live-streamed media featuring sexual acts,” our scope extends beyond traditional studio-produced pornography to encompass live-streamed webcam videos, pornographic content sold on platforms like OnlyFans, filmed conventional (in-person) prostitution, and recorded sexual abuse—covering a range of scenarios that are documented and disseminated as pornography.

Despite this broad definition, our theoretical starting point situates pornography as a global industry where vulnerable groups and individuals are disproportionately represented, and where “gender inequality is eroticized” (Swedish Public Health Agency, 2019, p. 158). In pornography, male violence against women is not only prevalent but is positioned as acceptable and even humorous (Bridges et al., 2010; Carrotte et al., 2020; Dworkin, 1981; Fritz et al., 2020; Purcell, 2012; Shor, 2019; Vera-Gray et al., 2021). With few exceptions (e.g., simulated sexual acts or in AI-generated pornography), this violence is inflicted on real women, with real consequences (Dworkin, 1981; MacKinnon, 1993). The intersection of pornography with prostitution has also led some scholars to theorize commercial pornography as a form of prostitution—one that engenders unique harms due to its documentation and dissemination (e.g., Dines et al., 1998; MacKinnon, 1993; Waltman, 2021).

This theoretical approach contrasts with an alternative view that emphasizes individual autonomy, framing pornography as a means to explore and express one's sexuality, derive pleasure, and earn an income simultaneously (Jones, 2016). From this perspective, platforms like OnlyFans are seen as empowering spaces where individuals—particularly women—can exert control over their content and engagement (Hamilton et al., 2022).

Acknowledging these diverse perspectives, our study, based in Sweden, aligns with the critical feminist framework, emphasizing the gendered power imbalances inherent in pornography. From this starting point, our aim is to explore and illuminate the lived realities and consequences of being filmed for pornography production.

## Method

The data collected were part of the larger research project “Exposure to violence and service needs among individuals with experiences in pornography production” commissioned by the Swedish governmental inquiry (Swedish Ministry of Employment, 2023a). The research, including both qualitative and quantitative elements, aimed to gain deeper insights into the situation faced by individuals filmed for pornography production, with a particular emphasis on those who had sought assistance from support services. Data collection and analysis were guided by teller-focused interviewing (Hydén, 2014) and reflexive thematic analysis (Braun & Clarke, 2006, 2022). Ethical approval was obtained from the Swedish Ethical Review Authority (reference number 2022-06718-01).

### Recruitment

We developed a recruitment ad detailing the study's purpose, target population, interview duration, remuneration, confidentiality, and contact information for the researchers. To increase the likelihood of reaching the target population, we used a variety of descriptors for pornography, for example being filmed for pornography by a third party, or selling self-produced sexual material (Ellard-Gray et al., 2015). The ad was distributed through relevant public agencies and civil society organizations in Stockholm and surrounding areas, and shared on social media to broaden the geographic reach. These stakeholders included psychiatric services for young people, youth and women's shelters, peer support networks, as well as clinics and specialized support organizations providing health services, legal assistance, counseling, outreach services, exit programs, and other forms of support for individuals with experiences in prostitution and other parts of the sex industry. Collectively, these services address a wide range of needs, including psychological well-being, sexual health, safety, legal aid, and recovery, and are accessible to anyone seeking assistance.

The inclusion criteria required participants to be 18 years of age or older and to have been photographed or filmed for pornography. Forty individuals responded to the recruitment ad and expressed interest in participating in the study. Those who met the inclusion criteria and wished to participate were invited to a face-to-face interview at a time and place of their choosing, either in-person or online. A total of 28 individuals were interviewed between March 26 and October 20, 2023.

### Data Collection

The narrative-focused interview method (Hydén, 2014), developed for research on sensitive topics, incorporates various strategies to establish a relationally safe space and address the power imbalance between the interviewer and the participant. For instance, participants choose the interview location to maximize their sense of safety and control. The interview process is also viewed as a collaborative effort, with the researcher and the participant having distinct roles and responsibilities, yet both contributing to the development of the narrative (Hydén, 2014).

Prior to each interview, informed consent was obtained both orally and in written form. A semistructured interview guide was used as inspiration for questions related to key topics such as childhood experiences, experiences of being filmed for pornography, and interactions with support services. While the interview guide served as a prompt for discussion, participants were given ample space to share their stories and discuss which experiences were most paramount for them. Each interview lasted approximately 60 minutes (Hydén, 2014).

Participants were provided contact details for the research team and available support services if they wished to receive support following the interview (Campbell et al., 2019). One participant followed up after the interview to be able to talk through some of their experiences. Participants characterized the research as *very* important, and many voiced that the interview was a positive and helpful experience. They were remunerated with 500 SEK—an amount chosen to reduce the power imbalance between the interviewer and participant, acknowledging the contributions and time commitment of both parties, while avoiding potential coercion (Coy, 2006).

The interviews were audio-recorded, and the sound files transferred to safe online storage. Most of the verbatim transcriptions of the interviews were made by an external company, and the remaining were completed by the first author. Confidentiality was ensured by using pseudonymized names for the material, recordings, and participant quotes.

### Participants

The 28 participants (average age 27.3 years, range 18–39; 26 women, 2 men) were primarily Swedish natives (93%). At the time of interview, 12 participants had full-time or part-time employment, four were students, four were on sick leave, and seven were unemployed—four of whom were receiving support at a safe house or group home. Fifteen identified as bisexual, eight as heterosexual, one as homosexual, one as pansexual, and three as “other.” Half of the participants were diagnosed with a neuropsychiatric disability, typically autism and/or ADHD. All female participants and one of the male participants reported being sexually abused as a child (96%). The average age at the first time of being filmed for pornography was 16.6 (range 6–32). Most had experiences of self-produced pornography sold on platforms such as OnlyFans (86%), and many had been filmed for pornography by third parties for financial gain (64%) and had intimate images disseminated without their consent (64%). Half of the respondents were filmed for pornography while being sexually abused, and half were filmed while selling sexual acts in conventional prostitution.

### Data Analysis

For the reflexive thematic analysis (Braun & Clarke, 2006, 2022), we adopted an inductive and experiential analysis of the data. This approach was deemed most suitable, as the study's aim was to provide a platform for the voices of this understudied group, prioritizing their own experiences, perspectives, and meanings. The analysis was guided by a broadly critical realist perspective, which holds that while an objective reality exists, perceptions of that reality are shaped by social contexts and constructs. In this study, the reality explored—what pornography represents for individuals filmed in it—is thus a situated and interpreted reality, embedded within a specific context (Braun & Clarke, 2022). Aiming to prioritize participant voices, our coding was primarily inductive and data-driven, without adherence to a predetermined theoretical framework or coding scheme. Our analysis included both semantic and latent features of the data, incorporating their conveyed meanings as well as our own interpretive analysis.

The first author conducted most interviews, and the last author conducted the remaining interviews together with a research assistant. All transcripts were read and reread for familiarization. Each transcript was then read closely, and any segments of the text potentially relevant to the research question were tagged with a code label. During analysis, themes were creatively generated through active engagement with the codes. The dataset was coded twice to ensure rigor, switching the sequence of transcripts during the second coding run, and further developing the codes as the analytic process evolved (Braun & Clarke, 2022, p. 79). Codes were then clustered into broader candidate themes, which were revised and finalized by the first and last author by revisiting codes and data excerpts, ensuring a good fit with the data. The involvement of the first and last author throughout the analysis was a form of investigator triangulation, providing validation, diverse perspectives, and added breadth to the findings (Carter et al., 2014; Polit & Beck, 2012). All authors reviewed the final themes and drafts of the manuscript.

#### Subjectivity and Reflexivity as a Resource

Throughout the research process, the research team strived to reflexively acknowledge how their background, theoretical lens, and engagement with previous literature influenced the research. Personal subjectivity was regarded as a resource (Braun & Clarke, 2022). For example, the first author drew on professional experiences supporting and building rapport with the study population at a shelter run by a Swedish non-profit organization. This, combined with the teller-focused interview method, contributed to helping participants feel at ease and in posing culturally sensitive and appropriate questions. We strived to remain curious, open to new findings, and prepared to challenge and reevaluate our own preconceived notions and theoretical assumptions.

## Findings

We developed three main themes during data analysis: *Coercion behind the illusion of desire*, *A public record*, and *Enduring impacts*, discussed in turn below.

### Coercion Behind the Illusion of Desire

The first evident pattern identified was the stark discrepancy between what the production of pornography is presumed to involve—desire, consent, sex—and what it in fact represented for participants. Far from being a manifestation of their own volition, their appearance in pornography represented an illusion of desire, dictated by overt and covert coercion.

Nearly all participants had experiences being filmed or photographed for pornography under direct duress. The abuse was perpetrated by a range of male perpetrators, including fathers, stepfathers, other male relatives, intimate partners, and traffickers. Typically, the first instance of filmed abuse was perpetrated during the participants’ childhood, or otherwise early adulthood. The abuse could represent a one-time violation that was recorded. Linnea, for example, was pressured into being filmed while engaging in sexual acts with her then boyfriend, who promised that the images would remain private. For her, the violation came when she discovered that the video had been uploaded by him months later to Pornhub—with his face blurred, her face fully visible.

For most, however, appearing in pornography represented not just one instance of filmed abuse but the documentation and dissemination of long-term, systematic exploitation. Several participants were first abused as young children by their fathers. Emma defined her childhood as “seeped in misery,” dominated by her father's tyrannical abuse. From an early age, she endured his physical, psychological, and sexual abuse, which included being sold to other men. These violations were first documented and disseminated on dark web websites, but later uploaded to global pornographic sites like Pornhub.

When I was about 6 years old, my Dad began filming everything he did to me. He would invite his friends over and let them exploit me, and he uploaded the videos onto different websites. … First dark web sites for videos of children. But as I got older, maybe around 13, 14, he made it seem like I was 18 and posted the videos to websites like Pornhub. And no one knew he was my Dad. (Emma)

The excerpt above underscores the stark contrast between the illusion of desire and the underlying reality of grave sexual crimes being committed against a vulnerable child. As Emma articulated, men who encounter these videos and images on websites like Pornhub remain oblivious to the fact that they represent documented incest and the rape and exploitation of a child.

Other participants were first exploited in pornography by male intimate partners, either as teenagers or as young adults. Sandra was secretly filmed at 18 by her partner, and later discovered to her horror that the images had been spread.

At one point I turned around and saw that he was taking a video. I asked him what he was doing! And I got really scared. He began to say, “Yeah, but you know that you can make money off of this?” Which it turns out he had done … it was awful. (Sandra)

Some were aware of the documentation but were forced to engage in sexual acts due to ongoing intimate partner violence and entrapment. Laura, for example, described how her abusive boyfriend would document the sexual violence he subjected her to.

(He filmed videos), but not with my consent. He put up the camera somewhere. … I didn’t want to be with him, but he forced me into a relationship. … He harassed and threatened me…he made me come over to his place every day and he controlled every aspect of my life…I was so scared of him. (Laura)

In several cases, intimate partners operated as traffickers, forcing victims to sell sexual acts to buyers, both online or offline. Alva recounted how her then-husband, who was physically, psychologically, and sexually violent, forced her to perform sexual acts for an account on OnlyFans. Although the account appeared to be run by Alva herself, it was fully controlled by her husband. Buyers, under the illusion they were interacting with Alva, were actually communicating with her husband—her trafficker. Alva also revealed how she was forced to present her experiences on OnlyFans in a positive light during telephone interviews with journalists. The journalists, completely unaware that her husband was sitting right next to her dictating her responses, unwittingly reinforced the illusion of Alvas willingness to appear in pornography.

The illusion of desire is indeed most apparent in such settings of tyrannical control and abuse. Victims appear compliant, but this compliance is a facade maintained under duress and the threat of further harm, functioning as a survival strategy to avoid additional violence. However, the illusion of desire does not solely manifest in the presence of direct force by third parties. The narratives reveal a subtler, yet equally forceful, form of coercion. Any form of payment creates a dynamic in which the buyer ultimately dictates the terms of the interaction, often at the expense of the seller's boundaries and well-being. This dynamic persists across various contexts—whether sexual acts are sold in person or through technology, in real time or otherwise—and regardless of who is paying, be it a pornography producer, a buyer on platforms like OnlyFans, or a buyer in conventional prostitution. Even in the absence of third-party control, payment acts as a coercive force, pressuring sellers of sexual acts to perform them not out of personal desire but to fulfill and cater to the fantasies and expectations of buyers and consumers.

While participants varied in how they interpreted their experiences—some expressing ambivalence or framing aspects as pragmatic trade-offs, and others describing them as unequivocal abuse—across accounts, the power imbalance created by payment was evident. For instance, Maria, who sold self-produced pornography to finance an addiction, described her inability to refuse the buyers’ demands, despite the degrading and harmful nature of the acts. She explained, “Everything happened on their terms, so it was incredibly degrading for me. I wasn’t in a position where I could control what I did, because I was in desperate need of money for the drugs.”

Jenny, who sold webcam pornography, explained that she had to perform the sexual acts listed on her “menu” to retain buyers. Oscar, who sold pornography on OnlyFans, described discomfort with certain requests from male buyers, noting that financial incentives made it difficult to refuse. The following excerpt highlights how the sexual acts he performed were not rooted in his own sexuality or volition but dictated by buyer's terms:

I wouldn’t have taken those pics if it weren’t for OnlyFans. … Being straight, I had my boundaries. There were times when I got a request for something I didn’t really want to do. But they offered a lot of money. So, I thought, “Okay, I’ll just film this video that I don’t actually want to do.” Send it. And then if someone asks for it again, I already have the video and don’t need to film it a second time. That was my rationale. If I could build up a library of stuff I didn’t want to do, then at least I wouldn’t have to suffer. But more requests came in for things I didn’t want to do. It was—hard to say no. (Oscar)

The coercive role of payment was perhaps most evident in Hanna's narrative, which contrasted sharply with the experiences of other participants. Unlike those who were first filmed for pornography under duress during childhood or early adulthood, Hanna began uploading filmed sexual acts with her boyfriend to a noncommercial platform in her 30s, where she could select who could access the images. Although she still faced the risk of the images being disseminated outside of her control, the absence of payment was paramount. Hanna explained that introducing payment would create external pressures from buyers, transforming the filmed sexual acts into ones performed solely to satisfy the specific desires and demands of the buyers.

The narratives reveal that coercion through payment compels more than just unwanted sexual acts; it also requires assuming a “fantasy role.” This role involves feigning enthusiasm for the sexual acts and fabricating sexual desire toward the male buyers, even when the seller feels repulsion toward these men. Indeed, participants described male buyers as men lacking empathy who exploit others’ vulnerabilities for personal gain. Alice articulated the profound psychological toll of adhering to the imposed fantasy role, demanding behaviors that starkly contradicted her own ethics and desires.

The toughest part is having to talk to them, and if you haven’t posted anything in a week, they’re like, “I’m going to cancel my subscription.” And people are always on your case, “Why aren’t you replying?” And they want to meet up. You have to keep up with the fantasy they have. … So, you can’t just say, “No, I never want to meet you. I hate you.” Instead, you have to play the role. (Alice)

Alice's assertion, “You can’t just say, ‘No I never want to see you. I hate you,’” reveals the inherent contradiction and illusion at the core of her experience. Economic coercion compelled her to mask feelings of disgust and repulsion with a façade of desire and willingness. Malin echoed this sentiment, emphasizing how buyers choose to believe in a fantasy far removed from reality: “It's super easy to play the part that they want to believe…that I’m there enjoying it, choosing to be there because it's sexual freedom. … That fantasy—it doesn’t exist, not for anyone.” Emelie explained how over time, this façade—present whenever sexual acts are performed for payment—can eventually become one's “normal”:

I know you guys…think these girls are doing it totally willingly because they’re pretending… But … you should always assume she's not there by choice. It's the same when buying someone in person. Anyone can pretend, and over time, pretending just becomes normal. (Emelie)

In grappling with the dissonance between compelled behaviors and true feelings, as well as exposure to multiple forms of violence, participants developed necessary coping mechanisms and forms of resistance. Dissociation, i.e., mentally disconnecting from the present moment, was a commonly used strategy. Vilma shared how this strategy, while helping her survive, had long-term consequences, including feelings of disconnection from her body and dissociative seizures. She recounted, “I dissociated a lot. I just shut down. I was there but not really. I think that's why I don’t remember much, because I just turned off. That's also why I don’t feel connected to my own body today, and why I have these episodes.” Drugs and alcohol were also used to numb participants from the sexual acts and the role they were compelled to perform. Olivia recounted how she relied on substances to distance herself from the situation: “Otherwise, I wouldn’t have been able to say the things I said (to the men).”

Another survival strategy was the subconscious division of self into two distinct identities: one embodying the authentic self, and the other, the role and persona tailored to buyers. Evelina said, “It was like having two separate selves—one when I’m selling myself and my ‘normal’ self.” Serving as a psychological shield, the alternate or fantasy persona takes on the brunt of violence and the roles demanded by the situation, all to protect the authentic self and remain emotionally “untouched” by buyers and consumers. The greatest emotional turmoil arises when buyers expect and demand any displays of intimacy, which threaten to breach the boundary protecting the authentic self. Evelina described how she would rather endure buyers’ physical violence than simulate emotional intimacy: “Sometimes, what I found almost the hardest … were the times … when they wanted it to be like a relationship. Wanting to make out and stuff. I feel like that's almost worse than when they’re violent because it gets … intimate in a really forced way.”

The need to adopt a persona to protect one's authentic self, along with other strategies to mentally and emotionally distance oneself, underscores the inherently coercive dynamics at play—dynamics that demand compliance and self-denial in the pursuit of fulfilling others’ fantasies.

### A Public Record

The second theme highlights how the documentation of the sexual acts and roles outlined above, while not altering their essence, introduces unique and additional consequences. Pornography transforms the illusion of desire and the sexual acts into a public record. The ramifications of pornography's public nature are significant and unfold into three subthemes: *Identifiability*, *The paradox of proof*, and *Perpetual male entitlement*.

#### Identifiability

This subtheme explores the distress arising from the risk of being identified and associated with the pornographic images one has been filmed in. Participants articulated a profound sense of powerlessness over the fate of the images—if and where they were spread, where they ended up, who could access them, and if they would resurface. Due to the online nature of pornography, they felt that further dissemination was inevitable. Even not knowing whether the images had been shared or not was a source of ongoing worry.

Central to these anxieties was the fear of being recognized and *“*outed,” i.e., publicly linked to the individual depicted in the pornographic images. Compared to conventional prostitution, where sexual acts are sold in-person to sex buyers and thus retain a degree of anonymity, pornography widely disseminates and exposes such acts to a public, potentially global, audience. Whether through visual recognition, breaches of personal information, or being openly singled out, pornography makes what might have remained anonymous and hidden into something public and identifiable. Such visibility engenders a perpetual state of vigilance, with participants constantly worrying about who might have seen the images and recognize them. Anna described the dread of being “found out,” not least when starting a new job:

It's really messed up a lot for me…because I feel like I can never relax. I never know who might recognize me or where the (images have) spread. I’m can’t shake this nagging feeling that they’re going to pop up somewhere. Like now, when I’m starting a new job, I have this uneasy feeling. Who knows (about them)? It's an awful feeling I live with all the time. (Anna)

Anxieties over public exposure are also elucidated in Linnea's account. She feared what recognition of her in the leaked images on Pornhub might mean for her career.

I’m going to be a teacher, so I’m terrified that some student or parent might come across the link. If feels like that would be the end of my career, at least at that school. And the rumors would haunt me for a long time after. … My career would be destroyed by one video. And even though it's not my fault that it was distributed and recorded, it's me who bears the consequences. He (the perpetrator) faces none. (Linnea)

While the distress of potentially being recognized by *anyone* was significant, the deeper fear for participants had to do with the prospect of being outed to and identified by those whose opinions and relationships matter most, such as family, friends, and colleagues. The dread of being discovered by one's community acted as a powerful leverage for perpetrators to exert control over their victims. Emma detailed how the men who subjected her to sexual exploitation and abuse as a child threatened to send the images to her school if she did not cooperate: “My dad's friends have been contacting me a lot. The same ones who did those things to me, filmed me and all, they’ve reached out wanting to meet up again. They even said, ‘If you don’t meet with us, we’ll send this to your school.” Others recounted how buyers coerced them into continuing to send images or meeting in person by threatening to distribute the images to family members and others.

The fear of being recognized and connected to the images was a profound and haunting burden in the participant's lives. It explains why, when they had any opportunity to influence the situation, they took deliberate steps to conceal their identities whenever possible. Participants often hid their faces and obscured identifiable features such as tattoos. Some even underwent significant changes in their appearance to further reduce the chances of being recognized as the individual depicted in the pornographic images. Adopting a different name, altering one's appearance, and creating an alternate persona became essential strategies for attempting to preserve anonymity and shield their private lives from the invasive reach of buyers and public exposure.

#### The Paradox of Proof

The accounts reveal how the documentation and dissemination of the sexual acts and the roles that participants were compelled to perform created a paradox. On the one hand, most of the images that participants appeared in constituted documented crimes and violence, including human trafficking, rape, and sexual abuse. Yet the documentation and dissemination of such violence, under the guise of “pornography,” renders the violence invisible. Instead of being seen and treated as evidence of the crimes endured, the images become “proof” that they “wanted it.”

According to participants, these misperceptions affect more than just the consumers and buyers of pornography; they permeate society at large. Rather than being reassured that one's community would recognize and acknowledge the violence and coercion they endured, they dreaded—and indeed experienced—that friends, family and colleagues would presume that the images were “proof” of their willingness. They feared that in the eyes of the beholder, the fantasy persona they had to perform, and the real person behind it, become one and the same. This is why self-produced pornography felt particularly damning. There was concern that, even more so than with other forms of pornography, images where they appeared alone would be perceived as undeniable evidence of their willing involvement. Moa described, “In a way, it feels harder, more emotionally taxing when I film myself. … At least when someone else is filming, I can say that I felt forced to do it, that I was scared. It would feel easier to say that it wasn’t my decision, and that I’m able to put the blame on someone else.” In addition to being presumed to have participated willingly, individuals filmed for pornography face victim-blaming attitudes. As Jenny elucidated, there is a common view that “they should have known what they were getting themselves into.”

There's very little sympathy among people who aren’t in the camming world. If someone records your video and then posts it on a bunch of sites, the reaction is like ‘Well, that's your own fault.’ ‘Yeah, that's what you get for doing this kind of thing.’ That kind of attitude seems to be quite common. (Jenny)

She continued, clarifying that there is a difference between a stranger holding such victim-blaming views, and someone whom she personally knows.

Okay, fine if some random person on the internet thinks that, but imagine if an employer feels the same way. What if they find out or see me? I’m not sure what would happen then. It creates a sense of worry about how it could affect me and affect others who have been involved in pornography. It makes me sad that there's no sympathy. Or there is, but many people lack it. (Jenny)

Fears about facing victim-blaming attitudes were not unfounded. Beyond encountering these attitudes in media and other forums, participants disclosed first-hand experiences of being vilified and ostracized when the images they were featured in were discovered. Malin experienced the personal and financial consequences of being outed when she lost her job. Laura recalled being publicly exposed and scorned by teenage peers in her hometown—an action so psychologically painful that it led to a mental health crisis: “Someone tagged my private Instagram in a story, saying I offer certain services. Everyone in my small town saw it. After that, a lot of people turned on me. They called me all sorts of slurs, insults. … I was in a poor state mentally and ended up in the psych ward. It was a tough time for me.”

Widespread victim blaming and its ramifications were felt beyond the immediate circles of family, friends, peers, and colleagues. Participants described betrayal from the institutions and individuals formally tasked with providing protection and support. For example, after being subjected to a filmed gang rape, which was then spread on the internet, Ida turned to the Child and Adolescent Psychiatry Emergency Service (BUP-akuten) in hopes of finding support. However, the staff minimized the violence she had endured: “I went to the emergency psych services and talked to them. I’ve got to say, their response was awful. ... They said that what I went through was normal and that some people like that sort of thing. Totally dismissed me.” Ida's experience underscores a systematic issue wherein both individuals and institutions fail to recognize technology-facilitated sexual violence as violence. This failure is echoed in participants’ accounts of their attempts to report the violence to the police, frequently met with a dismissive attitude and that cases were not pursued with due diligence. They often faced a belittlement of their experiences and found that their cases had been hastily dismissed or dropped. At the core of these experiences was the misguided assumption of voluntary participation and victim-blaming attitudes.

#### Perpetual Male Entitlement

Beyond the loss of reputation, relationships, and employment, pornography's public nature jeopardizes any attempts to shield one's private life from the invasive reach of male buyers and consumers. The narratives reveal the sense of entitlement certain men can feel over the individuals, especially women, they see and interact with in pornography. Seen as products and dehumanized as “whores,” the men treat them as if they are perpetually available not only online, but also offline. For example, Julia, who was filmed when she was trafficked and sexually exploited as a child, shared how she was approached in a restaurant by a male stranger who had recognized her. The man felt not only entitled to expose her in public, but also assumed that he had the right to her body.

I was sitting with a friend at a pizzeria when this guy came in. He started to whistle at me. … I didn’t recognize him—I had no idea who he was. Then he asked if I wanted to follow him out, and I was like, “No, I’m here with my friend. I don’t want to go anywhere with you.” But then he showed me his phone, and right there in front of my friend, he said, “This is you, isn’t it? You do this kind of thing.” And then—my friend stopped being my friend because she thought I was a whore. (Julia)

Julia's painful experience illuminates the belief held by certain male buyers and consumers—namely that women featured in pornography are accessible, possessable, and devoid of the right to decline. This pernicious belief manifests in various forms of online and offline harassment, including subjection to “doxing,” the release of private information, or “sextortion,” the threat of releasing images. Anna, for example, recounted being stalked by a man who threatened and harassed her over the span of almost 2 years.

Yeah, I’ve been outed. I had a stalker who…got totally obsessed and threatened to expose me to my parents. He came to my house, just showed up uninvited, and it was really tough to deal with. He kept messaging me on Facebook. It was that classic move where he tried to ruin my life because he couldn’t be with me. He continued to harass me in various ways for almost two years. (Anna)

The public nature of pornography, combined with men's entitlement, creates a serious threat to personal safety. Evelina's account highlights the perils introduced by this dangerous concoction. Knowing the potentially life-threatening danger posed by male buyers, she was extremely vigilant in concealing her personal information and identity when she sold sexual acts, both in offline and online settings. However, through the public circulation of her images, men were eventually able to track down her personal identity. She was contacted on her private social media accounts by these unknown men, who threatened to harm her if she didn’t submit to their demands.

I was contacted privately on Facebook and Instagram, and I think on my phone too, by a handful of men who wanted to meet me. … And then I also received threats from one person who I was more scared of because I know the violence he's capable of. He wrote that he was standing outside my doorstep and sent the names and numbers of my parents’ names, and the names of my siblings. He wrote really disturbing things. It was really unsettling, from always feeling very anonymous, to them figuring out exactly who I was, where I lived, my friends. They somehow tracked me down. (Evelina)

Beyond the direct threat to safety, the experience of being identified and outed endangers the crucial psychological separation between the fantasy persona and the authentic self. Once Evelina's personal identity became known to buyers, her carefully maintained boundaries collapsed, leading to a sense of hopelessness and resignation. She felt that she could no longer protect herself—physically and psychologically—from the men's invasive reach.

For a long time, I was really good at keeping two separate selves. There was “me” when I sold myself, and there was the regular “me.” … But towards the end, it all started to merge. … After my identity was exposed, it seemed like it didn’t matter anymore because everything was ruined anyway. That's when I brought one or two sex buyers to my apartment, something I would have never done before. But I mean, like they said: “What does it matter now when we know where you live?” (Evelina)

While the presence of a camera does not alter the essence of the act being recorded, its documentation and circulation introduce additional threat by the men who believe they are entitled to the individual appearing on their screen.

### Enduring Impacts

In this theme, we examine how pornography's online nature creates lasting repercussions for those who appear in its content, affecting their mental health and efforts to reintegrate into society.

Participants reflected on the difficulty of locating all the digital images they appeared in and the challenge of removing them. Many actively scanned the internet for their images and tried to remove them, only to find that they resurfaced or had not been taken down.

There are still pictures and videos of me being uploaded today. I can find them if I search my username, and I’ve even tried using a facial recognition service, which brings up a bunch of sites. I have no idea what to do, because it's not easy to remove them…It's super complicated, and there are so many websites. It feels like they’re automatically re-uploaded. So, it's pretty tough. (Jenny)

Some, aware of the challenge of removing the images, reached a reluctant acceptance that the images might remain online indefinitely. They hoped that with time, the risk of being associated with the person appearing in the images would diminish. For most participants, however, the perceived permanence of pornography was described as an enduring burden, permanently linking the individual with the illusion they had been compelled to perform. The risks associated with pornography as a “public record”—including being recognized, outed, and potentially ostracized or dehumanized—were not temporary concerns but persistent threats that created ongoing vulnerability and psychological distress. Anna described, “The material doesn’t go away. And there's this constant anxiety of wondering who has seen it … and when you might get outed and so on. It's a really brutal kind of anxiety to carry around.”

The perceived permanence of pornography further distinguishes it from conventional prostitution. Moa reflected on how, initially, the presence of a camera felt no different from situations without one. Yet, over time, the profound implications of having such acts documented and disseminated became increasingly clear.

It can feel like it doesn’t matter, like “I’ve already lost a part of myself, what the hell, what difference does it make?” But even if you don’t think about it in the moment, the consequence of being filmed—it's like adding a weight to the heavy backpack you’re already carrying. It becomes an extra burden because it might never disappear, and you don’t know what will happen with it. … There's a big difference between things happening behind closed doors and things being out there for everyone to see. … Knowing that the things that happened are somehow forever, is really tough. It's as though a part of me will never escape because [the images are] still out there. (Moa)

For those seeking to exit the sex industry, the enduring nature of pornography can undermine their efforts to feel completely free from one's past. This perceived permanence creates a profound sense of entrapment, making them feel unable to ever fully “escape.” Julia, for example, described how instances of public recognition inexorably pulled her back to the traumatic past she was striving to leave behind: “…people can still say, ‘I recognize you, I’ve seen you.’ And in that instant, it's like I’m transported right back.” Even after several years of comprehensive, trauma-informed support, Julia still felt like she would never fully recover and feel free from her past. For both Julia and Wilma, however, recognizing that they had been exploited as children was a turning point in their recovery. Julia said, “I think it helps me to remember that I was under 18, and even under 15 at the time. ... I've started to look at it from a legal perspective, like, okay, it was a serious crime. It makes it just a little bit easier.” By understanding that the responsibility lay solely with their abusers, Julia and Wilma were able to shift some of the blame away from themselves, offering partial relief from the profound shame and self-blame they felt.

Evelina articulated how the imminent threat of being identified, combined with the dehumanizing views of women in pornography, deepened this sense of entrapment: “…it felt hopeless, like I could never be free, when they wrote things like, ‘Once a whore, always a whore.’” This assertion reflects the dangerous and damning belief that women and others in the sex industry are permanently possessable—a belief that has long-lasting, deleterious effects in the lives of those targeted.

The long-term impact of pornography on participants’ daily lives manifested in a range of severe health challenges, including substance abuse, multiple diagnoses such as PTSD, the need for medications, chronic health conditions, nonfatal self-injury, suicidal ideation, and suicide attempts. Ida's narrative vividly captures the depth of the psychological toll. She struggled to manage her education and ultimately relied on strong antidepressants to cope after the filmed group rape was disseminated online. She credited the medication with saving her life:

…the first year of high school was awful, I missed school almost all the time. I couldn't go to school for the full day. I imagined [the images] everywhere. I saw them in front of me all the time. I still often break down. I have my really strong medication. It's really my lifesaver. Because I don't know, it sounds really strange, but I don't know if I would be here without the medication. Because otherwise I would have tried to take my own life, and I'm 100% sure of that. If I didn’t have this to help me. And I still use them. And my mom can tell when I need them. (Ida)

Jenny, reflecting on her experience of selling webcam pornography, emphasized how the lasting toll on her health remains profound and irreversible: “I wish I could go back in time and make a different choice. The negative impact it's had on my well-being and my life—it's going to stay with me forever.”

These enduring impacts underscore the urgent need for robust support systems and comprehensive legal frameworks to address the unique and pervasive harm caused by the documentation and dissemination of sexual violence.

## Discussion

This study provides a unique exploration into the lived experiences of those filmed for pornography, examining its meanings and implications for their lives. While our sample primarily consisted of individuals who were in contact with or followed social media accounts of support services, it still captured a diverse range of experiences and perceptions about pornography, including both critical and somewhat positive views. Despite these differences, our findings consistently reveal that pornography represented first and foremost an illusion of desire. Whether through overt force, such as child sexual abuse or filmed rape, or through monetary coercion, participants described how they were compelled to perform and feign desire, sidelining their own needs and volition to satisfy the demands of male buyers and consumers. To cope, they employed various strategies to mentally and emotionally disengage and distance themselves from the filmed sexual acts—coping mechanisms previously identified in research among women in prostitution (Borg et al., 1981; Høigård & Finstad, 1992; Ross et al., 2012).

Understanding this illusion and the absence of genuine desire is crucial, as it directly ties to feminist critiques of common interpretations of consent. Traditional, legalistic definitions that equate consent with simply “granting permission” often fail to account for the power dynamics and complexities of coercion (Anderson, 2022). To address these power imbalances, feminist scholars have increasingly emphasized the importance of desire in understandings of consent (Anderson, 2022). For instance, philosopher Ellie Anderson (2022), for instance, argues that consent should be redefined as “feeling-with” or “desiring-with” another person, positioning sexual encounters as collaborative processes rooted in shared desire and the mutual unfolding of erotic experience. In contrast, the absence of mutual desire in our participants’ experiences transformed their encounters into unilateral acts rather than mutual ones (Coates & Wade, 2004). Participants described not merely a lack of desire but a profound sense of loathing and repulsion toward the buyers they were coerced—through force, manipulation, or financial need—to pretend to desire. This dissonance between their outward actions and inner feelings imposed a significant emotional and psychological toll.

Payment played a pivotal role in this dynamic, introducing a coercive force that pressured participants to override their boundaries and engage in acts dictated by buyers. Financial need, especially when compounded by systemic vulnerabilities such as poverty, gender inequality, and histories of abuse, left participants little choice but to comply. In contrast to the concept of reciprocal, mutual desire, which forms the foundation of genuine consent, the dynamic created by payment reflects an exploitative hierarchy where one party's gratification is prioritized over the other's autonomy and well-being.

Our findings diverge from Jones’ (2016) conclusion that the physical safety created through webcam pornography “usually” allows for mutual sexual pleasure between the seller and the buyer. However, an online forum where both sellers and buyers interact is an environment unlikely to eliminate the compelled roles and illusion of desire evident in the narratives. Rather, women in these contexts likely feel pressure to communicate in ways that align with the expectations of male buyers, making it difficult to engage in meaningful discussions about their realities. In contrast, in private community forums or in the context of in-depth research interviews, individuals may feel more comfortable to share a fuller, more nuanced account of their experiences.

Our results more closely align with the insights from the 1977 government-initiated inquiry on prostitution in Sweden (Borg et al., 1981; Månsson, 2017). Through fieldwork and in-depth interviews with women in prostitution, Hanna Olsson, chief secretary of the inquiry, revealed a paradox: despite assumptions that these women had a heightened affinity toward sex, their actual experiences in prostitution were markedly nonsexual. For them, prostitution was about catering to the buyer's sexuality and fantasies. Olsson argued that this dynamic epitomizes the traditional female role within a patriarchal society, where women are denied their own sexuality and must satisfy men's sexual fantasies at the expense of their own well-being (Borg et al., 1981, p. 296; Olsson, 2006, p. 20). Such insights were key in framing prostitution as a form of men's violence against women in Sweden and establishing the “Nordic” or “Equality model.”

The parallels between Olsson's analysis of prostitution and our present findings support the notion that pornography involving payment is the technology-facilitated version of conventional prostitution, an argument presented by Tyler (2015), among others (e.g., MacKinnon, 2004; Waltman, 2021; Whisnant, 2016). Indeed, consistent with other research on online sexual violence (e.g., Rindestig et al., 2023), participants in our study perceived the sexual acts filmed for pornography as largely indistinguishable and inseparable from their offline, nonrecorded equivalents. While not altering their fundamental nature, the use of digital technologies introduces unique and additional harms (Henry & Powell, 2018; Tyler, 2015). Pornography transforms these acts into a public record—not only used as masturbation material, but also risking the victim's identification, and thus physical and psychological safety, leaving lasting impacts on their lives.

Our findings reveal how documented sexual violence is often misinterpreted as proof of consent rather than as evidence of a crime, further compounding the victim's shame and suffering. This paradox was also highlighted in research among adult survivors of child pornography (Gewirtz-Meydan et al., 2018). In the study, several participants described how the widely recognized seriousness of the crime they were subjected to helped them to eventually understand that someone else was accountable for their suffering—thereby reducing their shame and self-blame. Our findings mirror these results with one critical caveat: while child pornography (i.e., child sexual abuse images) typically receives the grave response it deserves, society tends to overlook the abuse and exploitation in pornography once individuals reach adulthood.

### Legal and Societal Implications

Our findings highlight the need to reassess the pornographic content consumed by most men and boys, often on a daily basis (Donevan et al., 2021; Swedish Public Health Agency, 2019). This content is frequently perceived as consensual and devoid of coercion—an assumption the pornography industry has a vested interest in maintaining. Our findings raise serious concerns that the material used for men's sexual gratification is often the product of sexual violence, documented and disseminated for certain men's financial gain (i.e., “made for men by men”). Although our study does not encapsulate the experience of all individuals featured in pornography, it challenges the prevailing belief that individuals featured in these images, even those who appear to participate willingly, in fact do so, devoid of coercion.

The discrepancy between consumer beliefs and the actual experiences of those on the other side of the screen may be particularly pronounced for self-produced pornography, such as material sold on platforms like OnlyFans. Our results show that these images can still be produced under duress, with perpetrators remaining invisible to buyers and consumers. Moreover, irrespective of third-party involvement, the coercive role of payment compels individuals to conform to the expectations and demands of male buyers. The parallels drawn by Whisnant (2016) between women filmed for pornography and torture victims also emphasize the severe psychological impacts when individuals must participate in their own abuse—harms that are further exacerbated by the documentation and widespread dissemination of that abuse.

Our findings also underscore the necessity of acknowledging online and technologically-facilitated sexual violence. Earlier research among children in Sweden, which established the equivalence of online and offline sexual abuse (Jonsson & Svedin, 2017), influenced the Swedish legal approach to treat online rape and sexual abuse as seriously as offline violations. Despite growing recognition of prostitution as sexual violence (e.g., Alsalem, 2024; European Parliament, 2023), a significant gap remains in addressing its technology-facilitated counterpart. In an effort to address this discrepancy, a recent Swedish governmental inquiry has proposed broadening the scope of laws penalizing buyers and third parties to encompass technology-facilitated forms of prostitution (Swedish Ministry of Justice, 2023).

Beyond legal ramifications, our findings highlight the importance of advancing support and care for individuals exploited in pornography production. This support must address not only the sexual violence that was filmed but also the unique harms arising from its documentation and dissemination, including necessary content removal and protection from the invasive reach of men who perceive victims as mere “products” to be consumed. Encouragingly, two recently published government inquiries in Sweden—on exit programs for victims of prostitution (Swedish Ministry of Employment, 2023b) and improved support and care for individuals abused in pornography (Swedish Ministry of Employment, 2023a)—provide an extensive list of measures to begin to address the multifaceted consequences faced by survivors of sexual violence and exploitation. Recent inquiries into the pornography industry conducted in France and the United Kingdom (All-Party Parliamentary Group on Commercial Sexual Exploitation, 2023; Chrisafis, 2023; Haut Conseil à l’Egalité entre les femmes et les hommes, 2023) show a promising international shift toward implementing similar measures.

Finally, to combat victim blaming and the “paradox of proof,” our findings highlight the critical need for widespread educational measures to improve societal awareness of the coercive circumstances influencing appearance in pornography. An accurate understanding and response to pornography are only possible when accounting for the context in which pornography is created, how it is documented and disseminated, and the direct and broader implications of its use (Donevan, 2025).

The main limitation of this study is that our recruitment strategy primarily attracted individuals who are in contact with, or have encountered, service providers either in person or on social media. This approach likely emphasizes narratives from individuals who have been subjected to harm and sought help, potentially underrepresenting those with less adverse experiences. However, it is important to also acknowledge that some individuals who do not seek support may be among the most vulnerable, controlled by traffickers that restrict their ability to access assistance. As such, our findings may not fully capture the experiences of those who have not sought support, whether due to less perceived harm or ongoing control by exploiters.

Despite these limitations, our sample included a diverse range of experiences with various forms of pornography, differing levels of current involvement, and varying perspectives, which may enhance the relevance and applicability of our results to other groups and contexts. Furthermore, in light of recent research (e.g., Donevan, Jonsson & Svedin, 2025; El-Khoury Lesueur et al., 2024), governmental inquiries (e.g., All-Party Parliamentary Group on Commercial Sexual Exploitation, 2023; Haut Conseil à l'Egalité entre les femmes et les hommes, 2023; Swedish Ministry of Employment, 2023a), criminal investigations (e.g., Chrisafis, 2023), lawsuits (e.g., Marshall et al., 2024), and survivor accounts (e.g., Kristof, 2020; So et al., 2024), coercion and exploitation emerge as systemic rather than isolated phenomena in the pornography industry. Finally, the parallels between the present research and previous insights related to prostitution further reinforce the broader applicability of the identified “illusion of desire” and the coercive role of payment across various forms of commercial sexual acts.

An additional limitation is that most participants in our study were Swedish natives. To gain a more comprehensive understanding of this global issue, future research should focus on more diverse populations and contexts, including individuals with immigrant backgrounds and transgender people, as these groups may face pronounced victim blaming and barriers to support and protection.

In conclusion, this study sheds light on the overlooked experiences of individuals filmed for pornography production, revealing profound issues of coercion and exploitation, public exposure, and the serious and long-term impact of pornography on participants’ lives. These insights can inform public discourse and enhance legal and policy responses to protect and support those directly harmed by pornography.
